# Demographic Performance of *Helicoverpa zea* Populations on Dual and Triple-Gene Bt Cotton

**DOI:** 10.3390/toxins12090551

**Published:** 2020-08-28

**Authors:** Marcelo M. Rabelo, Silvana V. Paula-Moraes, Eliseu Jose G. Pereira, Blair D. Siegfried

**Affiliations:** 1 Department of Entomology, Universidade Federal de Viçosa, Viçosa 36570-900, MG, Brazil; eliseu.pereira@ufv.br; 2 Department of Entomology and Nematology, West Florida Research and Education Center, University of Florida, Jay, FL 32565, USA; paula.moraes@ufl.edu; 3 National Institute of Science and Technology in Plant-Pest Interactions, Universidade Federal de Viçosa, Viçosa 36570-900, MG, Brazil; 4 Department of Entomology and Nematology, University of Florida, Gainesville, FL 32608, USA; bsiegfried1@ufl.edu

**Keywords:** fitness, life table, cotton bollworm, corn earworm, toxin, resistance management

## Abstract

Insecticidal toxins from *Bacillus thuringiensis* (Bt) are valuable tools for pest management worldwide, contributing to the management of human disease insect vectors and phytophagous insect pests of agriculture and forestry. Here, we report the effects of dual and triple Bt toxins expressed in transgenic cotton cultivars on the fitness and demographic performance of *Helicoverpa zea* (Boddie)—a noctuid pest, known as cotton bollworm and corn earworm. Life-history traits were determined for individuals of three field populations from a region where *H. zea* overwintering is likely. Triple-gene Bt cotton cultivars that express Cry and Vip3Aa toxins killed 100% of the larvae in all populations tested. In contrast, dual-gene Bt cotton that express Cry1Ac+Cry1F and Cry1Ac+Cry2Ab allowed population growth with the intrinsic rate of population growth (*r_m_*) 38% lower than on non-Bt cotton. The insects feeding on Bt cotton plants that express Cry1Ac+Cry2Ab, Cry1Ac+Cry1F, or Cry1Ab+Cry2Ae exhibited reduced larval weight, survival rate, and increased development time. Additionally, fitness parameters varied significantly among the insect populations, even on non-Bt cotton plants, likely because of their different genetic background and/or previous Bt toxin exposure. This is the first report of the comparative fitness of *H. zea* field populations on dual-gene Bt cotton after the recent reports of field resistance to certain Bt toxins. These results document the population growth rates of *H. zea* from an agricultural landscape with 100% Bt cotton cultivars. Our results will contribute to the development and validation of resistance management recommendations.

## 1. Introduction

Transgenic crops that express insecticidal toxins from the bacterium *Bacillus thuringiensis* (Berliner) (Bt) provide valuable pest management options for pests of field crops worldwide [1,2,3]. Positive socio-economic and environmental impacts of Bt crop adoption have been reported since commercial release in 1996 [4,5]. In the United States (U.S.), the Bt technology provides control of the major cotton pests *Chloridea virescens* (Fabricius) (Lepidoptera: Noctuidae), *Pectinophora gossypiella* (Saunders) (Lepidoptera: Gelichiidae), and *Helicoverpa zea* (Boddie) (Lepidoptera: Noctuidae) [6,7]. Bt cotton also improves the management of other lepidopteran pests, such as *Spodoptera exigua* (Hübner), *Trichoplusia ni* (Hübner), *Spodoptera frugiperda* (J.E. Smith), *S. eridania* (Stoll), and *Chrysodeixis includens* (Walker) [8,9,10,11]. From 1996–2003, commercial Bt cotton in the U.S. was limited to events that expressed the Cry1Ac toxin [12]. Second generation dual-gene Bt cotton that expressed the toxins Cry1Ac+Cry2Ab, Cry1Ac+Cry1F, and Cry1Ab+Cry2Ae became available in 2003 and were widely adopted [7,13]. The goal of these second-generation cotton events is to reduce the risk of resistance evolution by targeting unique and independent target sites. Since 2014 the third generation of cotton events with triple Bt traits became available, including those expressing Cry1Ac+Cry1F+Vip3Aa19, Cry1Ac+Cry2Ab+Vip3Aa19, and Cry1Ab+Cry2Ae+Vip3Aa19. The Cry and Vip families are produced during different stages of the *B. thuringiensis* life cycle [14]. Vip3A shares no sequence homology with any known Bt Cry toxins. The amount of shared sequence homology between Bt toxins is an important indicator of the risk that the two Bt toxins will share binding sites on the midgut of the insect, predisposing the toxins to cross-resistance. Although the two toxin classes are thought to have a similar mode of action against the target insects, they have different receptors in the insect midgut [14,15].

The tobacco budworm, *C. virescens,* is highly susceptible to most commercial Cry Bt toxins, and Bt cotton usually reaches a high-dose condition, killing almost all heterozygotes for Bt resistance [8,16], even for single toxin events. However, the cotton bollworm, *H. zea* is less susceptible to Cry toxins expressed in cotton and corn, which, therefore, do not satisfy high-dose criteria [17]. Toxicological bioassays performed with populations of *H. zea* from the southeastern U.S., including populations from the Florida Panhandle, have indicated a decrease in susceptibility of *H. zea* populations to Cry1Ab, Cry1Ac, Cry1A.105, and Cry2Ab, but not to Vip3Aa [5,18,19,20]. However, a major resistance allele conferring high levels of Vip3Aa resistance in a field-derived strain of *H. zea* in Texas has been recently reported [21].

Field-evolved resistance in target pests is a threat to the success of the Bt technology, which may lead to control failures [22] and the need for traditional insecticides for supplemental control [23]. *Helicoverpa zea* exhibits a sequence of host crop utilization based on the temporal dynamics of the southeastern U.S. agricultural landscape. In general, the first generation of this pest feeds on Bt field corn, cultivated during the spring to the beginning of summer. Corn is a major host plant of *H. zea*. However, this pest has a low impact on the yield of field corn when it is planted early in the season [24]. Later in the summer, subsequent generations of *H. zea* disperse from corn to cotton, which becomes the prevalent Bt crop in the southeastern U.S. agricultural landscape until the end of the crop season. Thus, corn serves as a source of *H. zea* populations, and if these source populations develop on Bt corn, a dispersion of Bt pre-exposed survivors from corn to cotton is likely. Larval feeding in both crops producing the very same or similar Bt toxin exerts continuous selection pressure and raises concerns about the selection of resistant populations [13].

The United States Environmental Protection Agency (EPA) has listed the knowledge of pest biology and ecology as key elements in formulating a Bt insect resistance management (IRM) programs [25]. While complete studies documenting the reproductive potential of *H. zea* populations feeding on dual- or triple-toxin Bt cotton are scarce, Cry1Ab Bt corn, for which is only moderately toxic, might reduce *H. zea* growth potential [26,27]. Developing life tables for *H. zea* on dual and triple-gene Bt cotton events allow the documentation of key fitness parameters, such as survival, development time, fertility, and population growth [28]. Fitness components and rates of population growth (i.e., demographic performance), if available, contribute to developing models to predict the rate of resistance evolution in target pests or to comparatively assess different resistance management practices, such as the use of structured and/or natural refuges [22,29,30,31].

*Helicoverpa zea* populations from the Florida Panhandle represent valuable resources for documenting the fitness components and demographic performance of lepidopteran pests targeted by Bt toxins in cotton. The region is in the Gulf Coastal Plain of the southeastern U.S., an ecological transition zone between temperate and subtropical climates. In the region, *H. zea* populations can overwinter and disperse throughout the growing season [32,33]. The overwintering survival of *H. zea* could be a carry-over source of Bt resistance alleles for other regions and between seasons [13]. Furthermore, the region has a distinctive regional landscape consisting of natural vegetation, forests, and field crops. Cotton is cultivated in large areas (approximately 50,000 hectares), with 100% adoption of Bt cultivars [34,35]. In this study, we report individual and population fitness of representative *H. zea* larvae challenged with dual- and triple-toxin Bt cotton technologies, information that contributes to the development and validation of resistance management recommendations.

## 2. Results

### 2.1. Life-History Traits

The interaction between cotton cultivar and insect population was significant (Table 1, *p* < 0.05) for larval weight, larval development time, larval survival, and pupal development time. Pupal weight, pre-pupa time, and egg viability varied only with the main effects of either cultivar or population or both (Table 1), and the pupal viability did not significantly vary (*p* > 0.05).

The dual-gene Bt cotton cultivars significantly (*p* < 0.05) reduced larval and pupal weights in all populations tested (Table 2). The population from Escambia County exhibited the lowest larval weight on Cry1Ab+Cry2Ae and Cry1Ac+Cry2Ab relative to non-Bt cotton, while Cry1Ac+Cry1F had the least negative impact on larval and pupal weights relative to the other cultivars. All three dual-gene Bt cotton cultivars reduced larval weight equally in the Santa Rosa population compared to the non-Bt cotton. The population from Jackson had the lowest larval weight on Cry1Ac+Cry2Ab and both lowest larval and pupal weights on Cry1Ab+Cry2Ae, while Cry1Ac+Cry1F cotton did not impact larval or pupal weights compared to non-Bt cotton. Among populations, *H. zea* from Jackson had the lowest larval and pupal weights, even when feeding on non-Bt cotton. Regarding larval survival rates, the triple-gene Bt cotton cultivars caused 100% mortality of all populations tested, and therefore, were not included in further analysis of life-history traits (Table 3). *Helicoverpa zea* from Escambia and Jackson had similar larval survival on non-Bt and Cry1Ac+Cry1F cotton. However, larval survival was reduced on Cry1Ac+Cry2Ab and Cry1Ab+Cry2Ae. *Helicoverpa zea* from Santa Rosa had larval survival reduced by all dual-gene Bt cotton, with Cry1Ab+Cry2Ae resulting in the most severe reduction. Among dual-gene Bt cotton cultivars, Cry1Ac+Cry1F and Cry1Ab+Cry2Ae allowed the highest and lowest larval survival, respectively. The survivorship of pupa (i.e., pupal viability) ranged from 91 to 100% and did not vary among cotton cultivars or insect populations (Table 3). The larvae developed more slowly on dual-gene Bt cotton than on non-Bt cotton, except for *H. zea* from Escambia and Santa Rosa feeding on Cry1Ab+Cry2Ae (Table 4). The insects from Jackson county had longer larval development time when feeding on non-Bt and Cry1Ab+Cry2Ae than the other populations. The Santa Rosa insects had longer pre-pupa development time on Cry1Ac+Cry2Ab, but this trait was not affected by the other cultivars, populations, or their interaction (Table 4). The duration of the pupal stage was shorter for insects feeding on Cry1Ac+Cry1F than on non-Bt or the other Bt cotton cultivars (Table 4). The egg viability was similar in all-cotton cultivars, but was higher for the Escambia population (Table 5).

### 2.2. Life Table Parameters

**Figure 1:**
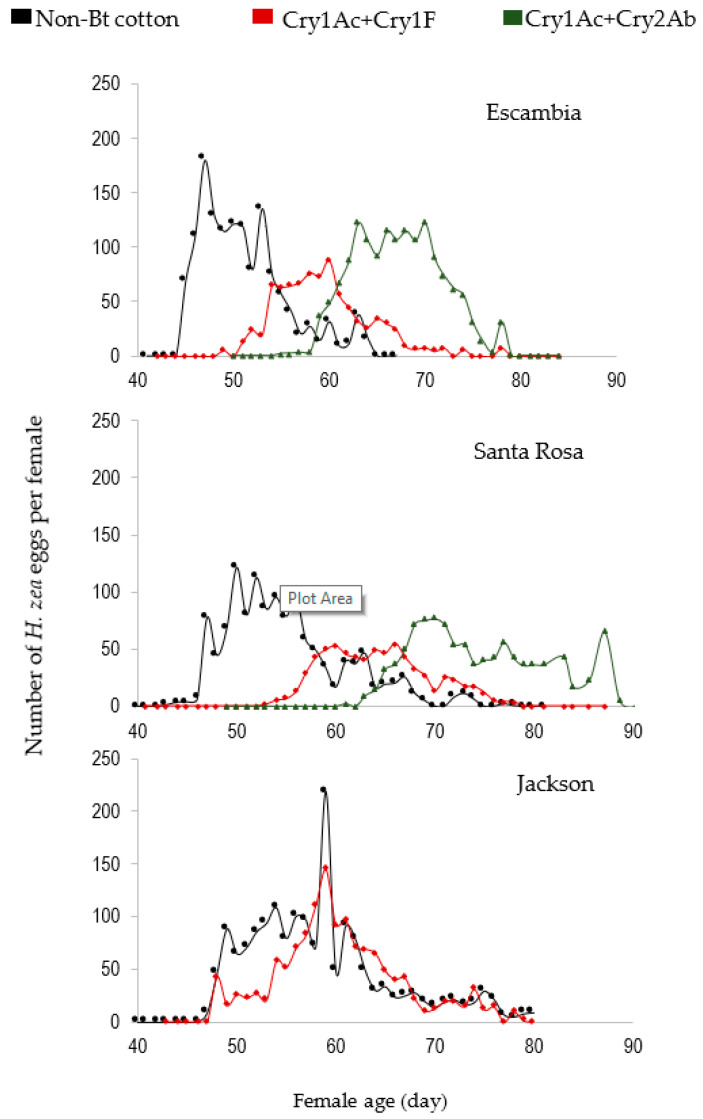
Reproductive schedule of *H.* *zea* feeding on non-Bt and Bt cotton cultivars as represented by fecundity (number of eggs per day) and female longevity. Each line represents an average of 16 *H. zea* females mated in pairs in mating cages. Panels A, B, and C represent the populations from Escambia, Santa Rosa, and Jackson county, respectively. The black line refers to insects feeding on non-Bt cotton, while the red and green are for insects feeding on Bt cotton Cry1Ac+Cry1F and Cry1Ac+Cry2Ab, respectively.

The demographic performance of *H. zea* feeding on non-Bt, Cry1Ac+Cry2Ab, and Cry1Ac+Cry1F varied among the cotton cultivars and insect populations (Table 6, Figure 1). Reproductive capacity on the other cultivars was not determined, due to low survival. The net reproductive rate (*R*_0_) of insects reared on non-Bt cotton was approximately 50% higher than those on Cry1Ac+Cry1F and Cry1Ac+Cry2Ab, except those from Jackson County, which exhibited the same *R*_0_ value on non-Bt and Cry1Ac+Cry1F. The intrinsic rate of population increase (*r_m_*) of the insects reared on non-Bt cotton was 30% greater than on Cry1Ac+Cry1F and Cry1Ac+Cry2Ab, except those from Jackson County, which exhibited the same *r_m_* value on non-Bt and Cry1Ac+Cry1F. The generation time (*T*) was nearly ten days shorter for insects reared on non-Bt cotton compared to those on the other cultivars, except for Jackson insects, which exhibited the same generation time on non-Bt and Cry1Ac+Cry1F. In contrast, the Jackson population had a higher fitness (higher *R_0,_ r_m_*, and lower *T*) on Cry1Ac+Cry1F compared to the others.

## 3. Discussion

In this study, the life-history traits and demographic performance of *H. zea* from the Florida Panhandle varied among cotton cultivars and field populations, indicating differences among the cultivars in the efficacy against *H. zea* and the current population susceptibility to the Bt toxins. Gassmann et al. (2009) suggest that survival, developmental time, and body weight are key individual fitness components [30]. Here, the effects on immature insect fitness components associated with both population and cotton cultivar translated to negative effects on the growth potential of *H. zea*. Although the non-Bt cotton cultivar used as control is not isoline of the Bt cultivars, the differences in the effects on *H. zea* life-history between the Bt and non-Bt cotton documented here are likely associated with the expression of Bt toxins in each cotton cultivar tissue. Cotton plants are rich in terpenoid compounds, which may function as a barrier against herbivores impairing growth/development and/or behavioral traits. However, the cotton plant has been modified during domestication and breeding for high yield and quality, including low gossypol oil in cottonseeds, which may have lessened the content of anti-herbivory secondary metabolites (such as gossypol) [36,37,38]. Information on secondary compounds in the cotton cultivars used in the present study was not available. However, other studies comparing non-Bt cotton cultivars have shown low or no change in noctuid life-history [39].

The triple-gene Bt cotton that expressed Vip3Aa (Cry1Ac+Cry1F+Vip3Aa19, Cry1Ac+Cry2Ab+Vip3Aa19, or Cry1Ab+Cry2Ae+Vip3Aa19) caused 100% larval mortality in all *H. zea* populations, which reinforces the high efficacy of this toxin for *H. zea* control [18,22]. These results suggest a low frequency of resistant alleles to Vip3A in *H. zea* populations tested. Data from laboratory and field in the U.S. consistently indicate high efficacy of the Vip3A against *H. zea* [6,40,41], and the debate has been focused on whether the trait meets the high dose definition [42]. Due to the relatively recent adoption of Vip3A toxins in commercial cultivars and limited insect sample size (40-130 individuals) in the present study, it would be unlikely to detect resistance to Vip3A at its current low frequency in the field. Vip toxins show limited amino-acid sequence homology with Cry toxins and cause pore formation with unique properties, thus, having a low risk for cross-resistance between them [14,43,44]. Cry toxins co-expressed in some Bt cotton cultivars have reportedly low impact in some *H. zea* populations [19,45], which compromises the pyramid of Bt genes. Our study demonstrates that life-history traits (body weight, survival, and development time) of insects from Escambia, Santa Rosa, and Jackson populations were negatively affected by the dual-gene Bt cotton cultivars that expressed Cry toxins.

Life-history traits were more affected by Cry1Ab+Cry2Ae and Cry1Ac+Cry2Ab, while Cry1Ac+Cry1F caused fewer negative impacts given the relative lack of toxicity that Cry1F has on the *H. zea* larvae and the widespread Cry1Ac resistance [19]. Significant mortality from Cry1Ac+Cry1F was observed only in the Santa Rosa population. Cry toxins have been expressed in Bt cotton cultivars since its first commercial release, and the first report of *H. zea* Cry1Ac resistance was documented 15 years later in the U.S., and recently the widespread resistance to Cry2Ab [6,20,46]. These may be a contributing factor to the considerable rates of larval survival on Cry1Ac+Cry2Ab and Cry1Ab+Cry2Ae cultivars. It also confirms the Cry1A and possibly Cry2A resistance alleles occurrence at high frequencies in the *H. zea* populations tested, which does not mean that the dual gene Bt cotton cultivars lost the benefit on *H. zea* management completely. Overall, our data on life-history traits (survival rates, body weight, developmental time) are consistent with previous reports that Cry1Ac+Cry1F affects *H. zea* larvae less than Cry1Ac+Cry2Ab, Cry1Ab+Cry2Ae, and cultivars expressing Vip3Aa [5,7,19].

Sublethal effects of Bt toxins on *H. zea*, as indicated by reduced body weight and the prolonged larval development, may have implications for pest management. Delayed larval development and low body weight are expected to increase the likelihood of exposure to other mortality factors. For example, early-instar larvae are unable to bore into the cotton bolls [47]. Consequently, they may be more exposed to insecticide applications and vulnerable to natural enemies [48]. Moreover, slow larval growth tends to increase the intervals for insecticide applications, which should target the most vulnerable stage of smaller larvae (about 1 cm) [49].

Cotton is the last summer crop to be planted in the Florida Panhandle region, remaining for a longer period than other crops in the agricultural landscape before the fallow season. The longer larval development time of *H. zea* when feeding on Cry1Ac+Cry2Ab, associated with infestations during mid- and late season, could expose larvae to shorter days and decreasing temperatures, factors that regulate insect diapause [50,51]. The Florida Panhandle is considered a “hybrid zone” of populations of noctuids, such as *S. frugiperda*, which flies from south Florida and Texas to the northern U.S. [52,53]. Diapausing and migration of *H. zea* populations from the Florida Panhandle may contribute to infestations in corn and cotton, North to 40 N latitude, where *H. zea* cannot permanently survive [32].

The pupal viability of *H. zea* was similar among different cotton cultivars and populations. In contrast, *H. zea* pupal weight varied when feeding on different cotton cultivars. Pupal weight is often correlated with fecundity [54], although this correlation might be affected by several other factors [55]. In our study, the heavier pupal weight was linked with higher fecundity, which agrees with reports for other noctuids, such as *H. armigera* [56].

Cumulative effects on specific life-history traits of *H. zea* (i.e., larval survival and development time) impact the population growth potential on Bt cotton cultivars. The life table parameters indicated that *H. zea* populations tested are expected to grow when feeding on Cry1Ac+Cry1F or Cry1Ac+Cry2Ab Bt cotton, but with reduced growth rates (i.e., *R*_0_, *r_m_*). Overall, insects feeding on Cry1Ac+Cry1F and Cry1Ac+Cry2Ab are expected to generate 30% and 42% fewer individuals per day compared to non-Bt cotton, respectively. The growth potential of *H. zea* from Jackson county population was similar when feeding on non-Bt or on Cry1Ac+Cry1F, which could result in a higher number of exposed offspring [12]. However, the insects of the Jackson county had lower growth rates on non-Bt cotton compared with Escambia and Santa Rosa populations, indicating the presence of fitness costs [57] when they do not feed on Cry1Ac+Cry1F cotton. In a theoretical scenario where only Cry1Ac+Cry1F cotton is cultivated in the Florida Panhandle, the *H. zea* population from Jackson county is expected to produce in one generation 10–27% more females per female than the populations from Escambia and Santa Rosa. These differences between *H. zea* populations reinforce that resistance may develop because of local selection [58,59].

During a period of over 23 years, in which commercialized Bt crops have been used in the U.S., IRM programs have relied on models to predict how quickly resistance to Bt may occur in different scenarios [60,61]. Although our study was performed in the laboratory and conclusions about field-evolved resistance are limited, this is one of the most complete life table studies of *H. zea* in Bt cotton available in the literature. The information provided in this study (life-history traits and life table parameters) can contribute to the refinement of predictive models and delayed resistance to important Bt toxins, such as Vip3Aa [22]. Our results reinforce the need for region-specific knowledge of target pests of Bt technology when designing IRM programs [60]. *Helicoverpa zea* has a high dispersal capacity and reproductive biology, which leads to extensive gene flow [60,61]. However, fitness components and their variability across environments should be taken into consideration in the simulation of predictive models [60].

In conclusion, this paper has quantified the dual and triple-gene Bt cotton effect on the life-history and demographic performance of three populations of *H. zea* from the Florida Panhandle. Triple-gene Bt cotton caused 100% larval mortality in all populations tested, indicating the value of Vip3Aa toxin on *H. zea* management in the region. Despite resistance, dual-gene Bt cotton containing Cry1A and Cry2A toxins significantly affected the fitness and demographic growth of the three populations of *H. zea* evaluated. However, the magnitude of the effect on the life-history, and consequently on the life table parameters of *H. zea* in a landscape containing 100% Bt cotton varied. Interaction between *H. zea* populations (Escambia, Santa Rosa, and Jackson counties) and cotton cultivars (Bt and non-Bt) was detected. These findings improve our understanding of how data on demographic growth rates of target pests to Bt technology matters and fill a gap by providing region-specific information when developing IRM programs. The results of this study also provide valuable parameters for the refinement of models to better predict the risk of resistance evolution and validate resistance management strategies, including refuge recommendations.

## 4. Materials and Methods

### 4.1. Cotton Plants

This study was conducted during 2018 at the West Florida Research and Education Center (WFREC), the University of Florida at Jay, FL, USA. The cotton cultivars utilized are adapted to the region and described in Table 7. The cultivars were planted in a Randomized Complete Block Design with four replications. Each cotton cultivar was planted on 5 m wide × 8 m long plots containing eight rows. The agronomic practices were based on standard recommendations for the region [62], and no applications of insecticides were performed in the experimental plots. Fully expanded cotton leaves were collected from the upper part of the plant canopy in each plot during the first bloom to open boll plant stages, placed in a ziplock bag (Johnson, Racine, WI, USA), and held in Styrofoam (ULINE, Chicago, FL, USA) boxes with an ice pack. In the laboratory, the cotton leaves were tested using Envirologix GMO quick Stix to confirm Cry1Ac, Cry2Ab, Cry1F, and Vip3Aa expression (EnviroLogix Kit, Portland, ME, USA) among the different events. Similar procedures were used to collect blooms, squares, and bolls at the early-middle stages of development also during the first bloom to open boll plant stages.

### 4.2. Insect Populations

Three *H. zea* populations were collected during the 2018 crop season from commercial fields located in the main cotton-producing counties in the Florida Panhandle: Santa Rosa, Escambia, and Jackson, USA. Cotton fields were located in areas where a peanut/cotton rotation is prevalent, with corn planted on a smaller scale. The populations from Santa Rosa (*n* = 100) and Jackson (*n* = 130) were collected from ears of Bt corn (Cry1A.105+Cry2Ab). The Escambia population (*n* = 40) was collected from blooms and bolls of Bt cotton (Cry1Ac+Cry2Ab). Information on the collections, including location, and the number of generations in the laboratory are shown in Table 8.

Collected larvae were identified based on their morphology and validated after adult emergence [63]. The larvae were removed from the plant individually and placed in plastic cups containing a multispecies lepidopteran diet (Southland Products, Lake Village, AR, USA). The cups were held in Styrofoam boxes with an ice pack during transport to the laboratory where they were maintained at 25 ± 2 °C, 70 ± 10% relative humidity and 14L: 10D photoperiod. Pupae were transferred to Petri dishes and covered with vermiculite moistened with water and placed in rearing cages (22 × 30 × 2.5 cm) for adult emergence. The adults were fed a solution of 10% honey which was replaced every two days. Paper towels (Great Value, Bentonville, AR, USA) were used to cover the internal walls of the cages as an oviposition substrate. The eggs were collected and transferred to ziplock bags until hatching. Neonates were transferred to a multispecies lepidopteran diet (Southland Products, Lake Village, AR, USA) in rearing containers (Southland Products, Lake Village, AR, USA) and maintained individually until pupation.

### 4.3. Life-History Traits and Life Table Parameters

One hundred *H. zea* neonates of each population (Santa Rosa, Escambia, and Jackson (Table 8)) were transferred in groups of five to 473 mL polypropylene containers (Fabri-Kal Corp. Kalamazoo, MI, USA) and fed with cotton leaves, blooms, squares, and bolls of the cultivars described in Table 7. After 5 d, the larvae were placed in individual containers to avoid cannibalism, as previously described [64]. The plant tissues were replaced every four days until pupation. When the larvae reached fourth instar, wet vermiculite was added to the bottom of the rearing containers as a substrate for pupation and to avoid desiccation. The pupae were left in the containers until adult emergence. Larvae weight was determined after seven days. Once larval development was completed, and within 24 h after pupation, each pupa was weighed, and the sex was determined. Other life-history components were recorded, including survival rate (neonate to pupa) and development time of larvae, pre-pupae, pupae, and adults. The experiment was arranged in a completely randomized design with 100 larvae per cotton cultivar (1 larva per replication) for each population.

One male and female from each cultivar that emerged within two days of one another were confined in mating cages (30 cm high × 20 cm diameter polymerized vinyl chloride tube). The cages were covered with a waxed brown paper (Roberts Consolidated Industries Inc., Boca Raton, FL, USA) as an oviposition substrate, and supplied with a 10% aqueous honey solution, replaced every day. Adult survival and the number of eggs were recorded daily. The brown paper containing the eggs was transferred to ziplock bags until hatching. An additional egg viability estimation was performed based on daily evaluation of the presence of the neonates in each ziplock bag. The sex ratio, number of eggs (fecundity), survival, and age of females at the onset of egg-laying were determined to estimate the life table parameters. These included the net reproductive rate (*R*_0_), which represents the multiplication rate per generation, the intrinsic rate of population increase (*r_m_*), which reflects the ability of one female to generate another female per unit of time, and generation time (*T*), the mean time between two successive generations. The life table experiment was conducted in a completely randomized design, with 16 to 18 replications (couples) per cotton cultivar.

### 4.4. Statistical Analyses

Differences in the survival rate, body weight, development time, and egg viability of the three populations reared on the seven cotton cultivars were compared using a two-way analysis of variance in R software (version 3.5.1) [65]. The fixed effects tested were the *H. zea* population, cotton cultivar, and their interaction. Pairwise comparisons were made using Tukey’s HSD (honestly significant difference) post hoc test using a level of significance of 0.05. The population growth parameters (*R*_0_, *r_m_*, *T*) were determined using the SAS programming developed by Maia (2000) [66], and the variances associated with the estimates were obtained by the Jackknife method. This procedure constructs confidence intervals for the estimated parameters in addition to comparisons by the *t*-test.

## Figures and Tables

**Table 1:** Two-way ANOVA for life-history traits of *Helicoverpa* *zea* populations feeding on cotton cultivars.

Variable	Source of Variation	*F*	*p*
Larval weight	Population	8.867	0.0003
	Cultivar	75.853	<0.0001
	Population × Cultivar	8.471	<0.0001
Larval development time	Population	12.959	<0.0001
	Cultivar	174.10	<0.0001
	Population × Cultivar	4.927	0.0002
Larval survival	Population	7.136	0.0010
	Cultivar	182.51	<0.0001
	Population × Cultivar	4.529	<0.0001
Pre-pupal development time	Population	0.99	0.3717
	Cultivar	5.18	0.0016
	Population × Cultivar	1.715	0.1302
Pupal weight	Population	9.43	0.0001
	Cultivar	15.70	<0.0001
	Population × Cultivar	1.58	0.1647
Pupal development time	Population	0.933	0.3943
	Cultivar	31.435	<0.0001
	Population × Cultivar	4.846	<0.0001
Pupal survival	Population	0.271	0.764
	Cultivar	0.691	0.561
	Population × Cultivar	0.520	0.760
Egg viability	Population	3.712	0.0275
	Cultivar	0.134	0.8751
	Population × Cultivar	0.129	0.9427

*p* values of 0.05 or lower were considered significant as calculated using two-way ANOVA in R software (version 3.5.1).

**Table 2:** Larval and pupal weight (mg) of *H. zea* reared on Bt and non-Bt cotton cultivars.

Stage	Population	Cotton Cultivar						
		Non-Bt	Cry1Ac+	Cry1Ac+	Cry1Ab+	Cry1Ac+	Cry1Ac+	Cry1Ab+
			Cry1F	Cry2Ab	Cry2Ae	Cry1F+	Cry2Ab+	Cry2Ae+
						Vip3Aa	Vip3Aa	Vip3Aa
Larva	Escambia	59.6 ± 24.4 Aa	25.5 ± 6.18 Ba	2.02 ± 2.14 Ca	0.29 ± 0.14 Ca	*	*	*
	Santa Rosa	56.8 ± 16.0 Aa	6.79 ± 1.96 Bb	2.31 ± 1.91 Ba	0.34 ± 0.17 Ba	*	*	*
	Jackson	20.1 ± 7.98 Ab	22.6 ± 6.03 Aa	0.26 ± 0.02 Bb	1.64 ± 1.66 Ba	*	*	*
Pupa	Escambia	429.0 ± 69.3 Aa	363.0 ± 49.7 Ca	393.0 ± 49.0 Ba	401.0 ± 00.0 Ba	*	*	*
	Santa Rosa	427.0 ± 67.7 Aa	386.0 ± 67.9 Ba	409.0 ± 40.4 Aa	324.0 ± 00.0 Ba	*	*	*
	Jackson	391.0 ± 70.7 Ab	373.0 ± 53.2 Aa	*	302.0 ± 80.8 Ba	*	*	*

Means (± SE) followed by the same capital letter within lines or the same lowercase latter within columns for each parameter do not significantly differ (*p* > 0.05; Tukey HSD). * not determined due to the high larval mortality.

**Table 3:** Larva and pupal survival rates (%) of *H. zea* reared on Bt and non-Bt cotton cultivars.

Stage	Population	Cotton Cultivar						
		non-Bt	Cry1Ac+	Cry1Ac+	Cry1Ab+	Cry1Ac+	Cry1Ac+	Cry1Ab+
			Cry1F	Cry2Ab	Cry2Ae	Cry1F+	Cry2Ab+	Cry2Ae+
						Vip3Aa	Vip3Aa	Vip3Aa
Larva	Escambia	93 ± 4.83 Aa	80 ± 0.7 Aa	30.0 ± 28.3 Ba	2.0 ± 0.4 Ca	0.0 ± 0.0 Ca	0.0 ± 0.0 Ca	0.0 ± 0.0 Ca
	Santa Rosa	83 ± 10.6 Aab	40 ± 1.0 Bb	18.0 ± 16.2 Bab	8.0 ± 0.8 Ca	0.0 ± 0.0 Ca	0.0 ± 0.0 Ca	0.0 ± 0.0 Ca
	Jackson	77 ± 16.4 Ab	80 ± 1.5 Aa	4.22± 2.00 Bb	13 ± 16.4 Ba	0.0 ± 0.0 Ca	0.0 ± 0.0 Ca	0.0 ± 0.0 Ca
Pupa	Escambia	96.9 ± 6.5 Aa	93.0 ± 11.4 Aa	95.2 ± 12.6 Aa	100 ± 0.0 Aa	*	*	*
	Santa Rosa	100 ± 0.0 Aa	96.3 ± 11.1 Aa	91.7 ± 20.0 Aa	100 ± 0.0 Aa	*	*	*
	Jackson	100 ± 0.0 Aa	95.9 ± 10.8 Aa	*	100 ± 0.0 Aa	*	*	*

Means (± SE) followed by the same capital letter within lines or the same lowercase latter within columns for each parameter do not significantly differ (*p* > 0.05; Tukey HSD). * not determined due to the high larval mortality.

**Table 4:** Development time (days) of *H. zea* reared on Bt and non-Bt cotton cultivars.

Stage	Population	Cotton Cultivar						
		Non-Bt	Cry1Ac+	Cry1Ac+	Cry1Ab+	Cry1Ac+	Cry1Ac+	Cry1Ab+
			Cry1F	Cry2Ab	Cry2Ae	Cry1F+	Cry2Ab+	Cry2Ae+
						Vip3Aa	Vip3Aa	Vip3Aa
Larva	Escambia	21.7 ± 1.43 Cb	28.3 ± 3.22 Ba	32.5 ± 4.79 Aa	21.00 ± 0.00 Cb	*	*	*
	Santa Rosa	21.9 ± 2.02 Cb	28.7 ± 2.89 Ba	32.9 ± 3.51 Aa	20.00 ± 3.50 Cb	*	*	*
	Jackson	24.3 ± 3.63 Ca	28.0 ± 3.18 Ba	*	37.2 ± 3.50 Aa	*	*	*
Pre-Pupa	Escambia	3.38 ± 0.71 Aa	3.45 ± 1.03 Aa	3.75 ± 1.08 Aa	3.00 ± 0.00 Aa	*	*	*
	Santa Rosa	3.52 ± 0.89 Ba	3.27 ± 0.84 Ba	4.36 ± 0.80 Aa	3.00 ± 0.00 Ba	*	*	*
	Jackson	3.48 ± 1.02 Ba	3.75 ± 0.91 Ba	*	2.75 ± 0.50 Aa	*	*	*
Pupa	Escambia	19.0 ± 1.05 Aa	17.4 ± 2.00 Ba	19.2 ± 1.18 Aa	21.00 ± 0.00 Aa	*	*	*
	Santa Rosa	18.4 ± 1.44 Ba	17.2 ± 2.78 Ca	20.9 ± 1.14 Aa	20.00 ± 1.81 Aa	*	*	*
	Jackson	19.3 ± 2.04 Aa	17.4 ± 2.00 Ba	*	17.00 ± 0.00 Ab	*	*	*

Means (± SE) followed by the same capital letter within lines or the same lowercase latter within columns for each parameter do not significantly differ (*p* > 0.05; Tukey HSD). * not determined due to the high larval mortality.

**Table 5:** Egg viability (%) of *H. zea* reared on Bt and non-Bt cotton cultivars.

Population	Cotton Cultivar						
	on-Bt	Cry1Ac+	Cry1Ac+	Cry1Ab+	Cry1Ac+	Cry1Ac+	Cry1Ab+
		Cry1F	Cry2Ab	Cry2Ae	Cry1F+	Cry2Ab+	Cry2Ae+
					Vip3Aa	Vip3Aa	Vip3Aa
Escambia	65.2 ± 37.6 Aa	59.0 ± 44.3 Aa	69.2 ± 34.2 Aa	*	*	*	*
Santa Rosa	44.3 ± 39.5 Ab	44.5 ± 33.9 Ab	43.8 ± 39.8 Ab	*	*	*	*
Jackson	43.3 ± 32.6 Ab	46.7 ± 36.6 Ab	43.3 ± 32.6 Ab	*	*	*	*

Means (± SE) followed by the same lowercase latter within columns do not significantly differ (*p* > 0.05; Tukey HSD). * not determined due to the high larval mortality.

**Table 6:** Life table of *H. zea* populations from different counties in the Florida Panhandle reared on Bt and non-Bt cotton cultivars.

Parameter	Population	Cotton Cultivar						
		Non-Bt	Cry1Ac+	Cry1Ac+	Cry1Ab+	Cry1Ac+	Cry1Ac+	Cry1Ab+
			Cry1F	Cry2Ab	Cry2Ae	Cry1F+	Cry2Ab+	Cry2Ae+
						Vip3Aa	Vip3Aa	Vip3Aa
* R_0_*	Escambia	320.62 ± 61.74 Aa	139.32 ± 18.99 Bb	133.54 ± 16.94 Ba	*	*	*	*
	Santa Rosa	406.80 ± 67.24 Aa	87.26 ± 12.71 Bc	80.57 ± 20.34 Ba	*	*	*	*
	Jackson	289.59 ± 53.32 Aa	270.09 ± 35.50 Aa	*	*	*	*	*
* r_m_*	Escambia	0.13 ± 0.006 Aa	0.10 ± 0.003 Bb	0.08 ± 0.003 Ca	*	*	*	*
	Santa Rosa	0.13 ± 0.006 Aa	0.08 ± 0.003 Bc	0.07 ± 0.005 Bb	*	*	*	*
	Jackson	0.11 ± 0.05 Ab	0.11 ± 0.03 Aa	*	*	*	*	*
*T*	Escambia	41.94 ± 0.67 Ab	48.88 ± 0.65 Bb	55.37 ± 0.65 Cb	*	*	*	*
	Santa Rosa	45.08 ± 0.95 Aa	52.99 ± 0.87 Ba	60.34 ± 1.73 Ca	*	*	*	*
	Jackson	47.62 ± 1.20 Aa	49.57 ± 0.75 Ab	*	*	*	*	*

Means (± SE) followed by the same capital letter within lines or the same lowercase latter within columns for each parameter do not significantly differ (*p* >  0.05) through pairwise comparisons using two-tailed t-tests after the jackknife method to estimate variance. *R*_0_—intrinsic rate of population increase (females per female per generation); *r_m_*—net reproductive rate (females per female per day); *T*—generation time (days). * not determined due to the high larval mortality.

**Table 7:** Non-Bt and Bt cotton cultivars, expressing *Bacillus thuringiensis* toxins, that were used in this study.

Trade Name	Cultivar	Family	Bt Event Name	Year Launched	Bt Toxin
Non-Bt	DP 1822XF	Deltapine	-	-	-
Bollgard II	DP 1646B2XF	Deltapine	MON15985	2003	Cry1Ac, Cry2Ab
WideStrike	PHY 444WRF	Phytogen	3006-210-23, 281-24-236	2005	Cry1Ac, Cry1F
TwinLink	ST 5122GLT	Stoneville	T304-40, GHB119	2014	Cry1Ab, Cry2Ae
Bollgard III	DP 1851B3XF	Deltapine	MON15985, COT102	2014	Cry1Ac, Cry2Ab, Vip3Aa19
WideStrike 3	PHY480W3FE	Phytogen	3006-210-23, 281-24-236, COT102	2015	Cry1Ac, Cry1F, Vip3Aa
TwinLink Plus	ST 5471GLTP	Stoneville	T304-40 × GHB119 × COT102	2017	Cry1Ab, Cry2Ae, Vip3Aa19

**Table 8:** *Helicoverpa zea* populations from the Florida Panhandle, 2018 crop season.

County	Geospatial Coordinate		Number of Insects Collected	Generation Tested
	Latitude	Longitude		
Santa Rosa	30.7757	−87.1432	100	F3
Escambia	30.9842	−87.4696	40	F2
Jackson	30.8041	−85.0805	130	F3
